# Differences in Life Space Activity Patterns Between Older Adults With Mild Cognitive Impairment Living Alone or as a Couple: Cohort Study Using Passive Activity Sensing

**DOI:** 10.2196/45876

**Published:** 2023-10-11

**Authors:** Marijn Muurling, Wan-Tai M Au-Yeung, Zachary Beattie, Chao-Yi Wu, Hiroko Dodge, Nathaniel K Rodrigues, Sarah Gothard, Lisa C Silbert, Lisa L Barnes, Joel S Steele, Jeffrey Kaye

**Affiliations:** 1 Department of Neurology, Alzheimer Center Amsterdam Vrije Universiteit Amsterdam, Amsterdam UMC locatie VUmc Amsterdam Netherlands; 2 Amsterdam Neuroscience - Neurodegeneration Vrije Universiteit Amsterdam Amsterdam Netherlands; 3 Oregon Center for Aging & Technology Oregon Health & Science University Portland, OR United States; 4 Layton Aging & Alzheimer’s Disease Research Center Oregon Health & Science University Portland, OR United States; 5 Department of Neurology Oregon Health & Science University Portland, OR United States; 6 Department of Neurology Massachusetts General Hospital Harvard Medical School Boston, MA United States; 7 Department of Neurology Portland Veterans Affairs Medical Center Portland, OR United States; 8 Rush Alzheimer’s Disease Center Rush University Medical Center Chicago, IL United States; 9 Indigenous Health Department University of North Dakota Grand Forks, ND United States

**Keywords:** passive monitoring, in-home sensor, mild cognitive impairment, 2-person home, life space activity, sensor, older adult, aging, elder, gerontology, geriatric, cognition, cognitive impairment, activity pattern, at home, daily activities, activities of daily living, digital health, old age, technology

## Abstract

**Background:**

Measuring function with passive in-home sensors has the advantages of real-world, objective, continuous, and unobtrusive measurement. However, previous studies have focused on 1-person homes only, which limits their generalizability.

**Objective:**

This study aimed to compare the life space activity patterns of participants living alone with those of participants living as a couple and to compare people with mild cognitive impairment (MCI) with cognitively normal participants in both 1- and 2-person homes.

**Methods:**

Passive infrared motion sensors and door contact sensors were installed in 1- and 2-person homes with cognitively normal residents or residents with MCI. A home was classified as an MCI home if at least 1 person in the home had MCI. Time out of home (TOOH), independent life space activity (ILSA), and use of the living room, kitchen, bathroom, and bedroom were calculated. Data were analyzed using the following methods: (1) daily averages over 4 weeks, (2) hourly averages (time of day) over 4 weeks, or (3) longitudinal day-to-day changes.

**Results:**

In total, 129 homes with people living alone (n=27, 20.9%, MCI and n=102, 79.1%, no-MCI homes) and 52 homes with people living as a couple (n=24, 46.2%, MCI and n=28, 53.8%, no-MCI homes) were included with a mean follow-up of 719 (SD 308) days. Using all 3 analysis methods, we found that 2-person homes showed a shorter TOOH, a longer ILSA, and shorter living room and kitchen use. In MCI homes, ILSA was higher in 2-person homes but lower in 1-person homes. The effects of MCI status on other outcomes were only found when using the hourly averages or longitudinal day-to-day changes over time, and they depended on the household type (alone vs residing as a couple).

**Conclusions:**

This study shows that in-home behavior is different when a participant is living alone compared to when they are living as a couple, meaning that the household type should be considered when studying in-home behavior. The effects of MCI status can be detected with in-home sensors, even in 2-person homes, but data should be analyzed on an hour-to-hour basis or longitudinally.

## Introduction

Mild cognitive impairment (MCI) is a syndrome characterized by impairment of 1 or multiple cognitive domains that is perceived to not cause major functional impairment in daily life [1]. By definition, people with MCI can function independently [2], that is, they do not have dementia. Half of those with MCI progress to the syndromic stage of dementia within 3 years [3], which means that functional decline worsens over time to the point where impairment clearly interferes with activities of daily living. To prevent loss of independence, which also leads to a high caregiver burden and high health care costs, improving function is thus an important target in clinical trials.

Functional decline is usually reported by people themselves or their informants, using questionnaires such as the Amsterdam Instrumental Activities of Daily Living Questionnaire (Amsterdam iADL-Q [4]), the Functional Activities Questionnaire (FAQ [5]), or the Clinical Dementia Rating (CDR [6]). These methods rely on recall or subjective interpretation of decline during a brief period (eg, “in the past week”), can only be administered periodically, and need active involvement of the participant and a partner, which can be burdensome. Further, the assessment does not consider whether the person lives alone or with a partner, nor does it consider the individual routine of the person with MCI or their coresident. As an alternative to address these limitations, remote monitoring technologies (RMTs), such as in-home sensors, can measure function objectively, continuously, and passively and in the home environment, meaning that no active involvement of the participant or their partner is needed while being observed in the real world.

Previous studies indicate that participants with MCI show altered activity levels and sleep patterns, as measured with wearables [7], and changing patterns of daily life activities, as measured with in-home sensors [8-10], compared to cognitively normal participants. However, many of these studies have a short measurement period or inclusion criteria that limit participation to those living alone. Additionally, studies that include participants living with a coresident may not fully consider the influence of one resident’s activity on the other. These factors can limit the interpretability and generalizability of the findings. With this context in mind, the aim of this study was to compare the everyday behavior activity patterns of older adults living alone with those of older adults living as a couple and to compare older adults with MCI with older adults who are cognitively normal using in-home passive sensors in both 1- and 2-person resident homes. Outcome measures included measures that can be calculated for both 1- and 2-person homes, such as room use, independent life space activity (ILSA), and time out of home (TOOH).

## Methods

### Recruitment

Participants from 3 cohorts in the Collaborative Aging Research Using Technology (CART) initiative were included: (1) a cohort (n=69 homes) from the Oregon Health & Science University (OHSU) of participants living in low-income, subsidized housing in Portland, Oregon (OHSU group); (2) a cohort (n=61 homes) of military veterans residing in the catchment area of the Veterans Integrated Service Network 20 (US Pacific Northwest), which included largely rural residing veterans (VA group); and (3) a cohort (n=51 homes) from the Rush University Medical Center (RUSH) of older African Americans (RUSH group) participating in the Minority Aging Research Study (MARS) [11]. Inclusion criteria for CART participants were as follows: (1) age≥62 years, (2) living alone (1-person home) or with a partner (2-person home), (3) absence of dementia, (4) not being wheelchair bound, (5) having current or willing to acquire internet access in the home, and (6) having basic technology knowledge (sending/receiving email) [11,12]. CART was a feasibility demonstration project where at least 60 participants per cohort site were planned for enrollment. The age of 62 years and above was chosen to be inclusive of younger older adults, including spouses. The low-income housing cohort was recruited via invitations to potential participants following presentations to several low-income (US Section 202 subsidized housing) facilities in the Portland metropolitan area. Veterans were recruited through community presentations to veterans’ groups and word of mouth among these veterans. The African American cohort was derived from the existing ongoing MARS study cohort in Chicago [13].

Unique to this study is that homes were treated as a unit, rather than looking at individual people living in those homes. A home was classified as an MCI home when at least 1 of the people living in that home was diagnosed with MCI at baseline. The “MCI home” label in this study did not change when the diagnosis changed during the study. The average age and education of a home were the average age and education of the persons living in that home.

### Study Protocol

Passive infrared (PIR) sensors (NYCE Sensors) were fixed to the wall of each room in every home. Door contact sensors (NYCE Sensors) were fixed to each door in each home, leading to outside the home to detect whether a door was open or closed. Initially, the sensors were installed for 1 year, but participants were asked to stay longer in the study once the sensors were installed. Weekly questionnaires were sent out to the participants asking whether in the past week any visitors stayed in the home for a night or more or whether the participants were away from home overnight.

A baseline visit and yearly follow-up visits included a neuropsychological assessment, physical and neurological examinations, assessment of cognitive status, activities of daily living, depression, anxiety, medical history and medication use, and life habits, as well as questionnaires assessing physical and mental health, loneliness, social activity, technology use, and function [11]. Participants were classified as having MCI based on a CDR global score of 0.5 [6] for the OHSU and VA groups. For the RUSH group, cognitive status was based on a clinical diagnosis by a neuropsychologist evaluating a cognitive assessment battery and a diagnostic classification by a clinician [14,15]. Basic demographic characteristics were collected for all residents in each home.

### Outcome Measures

**Figure 1:**
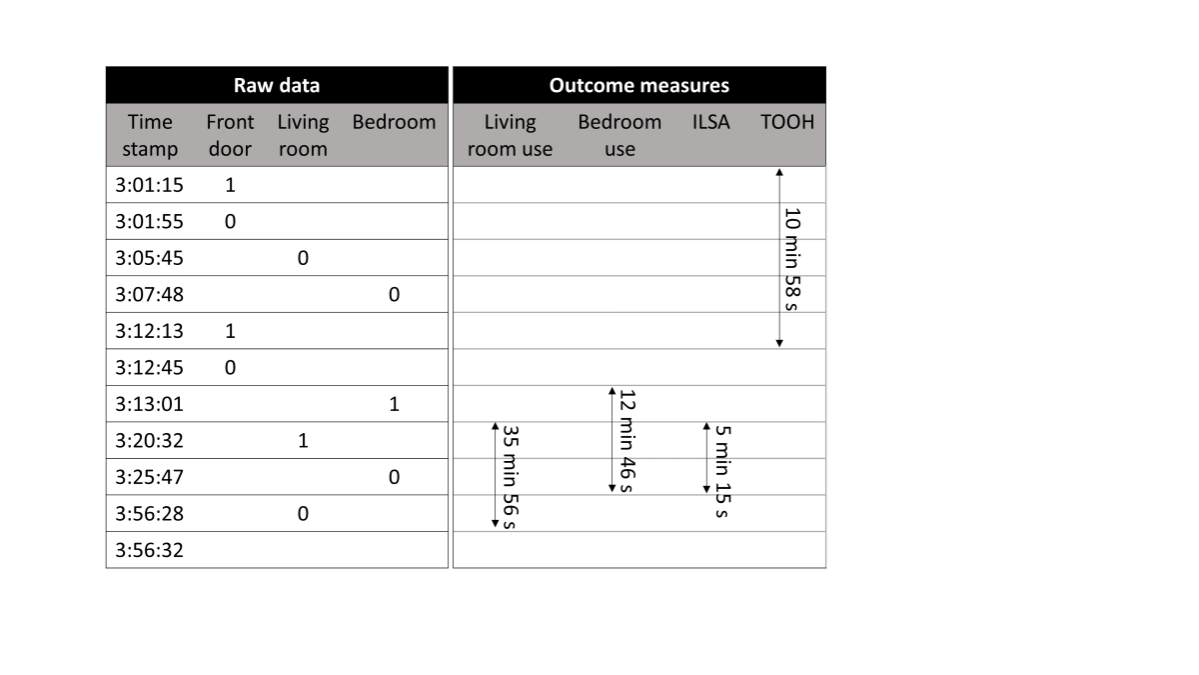
Outcome measures of in-home sensors. Here, 1 indicates door open (for door contact sensors) or motion detected (for room PIR motion sensors), while 0 means door closed (for door contact sensors) or no motion detected/motion no longer detected (for room PIR motion sensors). Room use was defined as the point from the moment motion was detected (1) to the moment motion was no longer detected (0), resulting in the living room being used between time stamps 3:20:32 and 3:56:28 (35 minutes and 56 seconds) and the bedroom being used between time stamps 3:13:01 and 3:25:47 (12 minutes and 46 seconds). The living room and bedroom were being used simultaneously between time stamps 3:20:32 and 3:25:47 (ie, motion detected in both rooms at the same time), resulting in ILSA being 5 minutes and 15 seconds. If there was only 1 person in the home who transitioned from the bedroom to the living room at 3:20:32, the bedroom motion sensor would have shown a 0 at time stamp 3:20:32. We therefore know for sure that there were at least 2 persons moving in the home. TOOH is the moment from time stamp 3:01:15 (door opens) to time stamp 3:12:13 (door opens after being closed), while no motion was detected in the rooms. ILSA: independent life space activity; PIR: passive infrared; TOOH: time out of home.

For this study, outcome measures were chosen that could be applied to both 1- and 2-person homes. Although, generally, PIR motion sensors detect only motion and cannot unambiguously differentiate between 2 persons, there are 3 conditions that can be extracted from the data with certainty: (1) when no one is in the home (no motion detected in any room between 2 door openings), (2) when there is at least 1 person in the home (motion detected in 1 room), and (3) when at least 2 persons are using 2 different rooms (motion detected in 2 different rooms at the same time). This resulted in the following outcome measures (Figure 1):

- Room use: Room use was calculated using PIR motion sensors. It was defined as the time of first motion detection by a sensor up to the time that motion was no longer detected by that sensor. Since PIR sensors detect motion as a change in the environment, sensors cannot detect motion when someone in a room is stationary (eg, when asleep). In 1-person rooms, it can be assumed that someone stays stationary in a room when no motion is detected in any other room in the home. However, when no motion is detected in a specific room in 2-person homes, there is no way to unambiguously verify whether a person has left the room or whether that person has stayed in the room without moving, since motion detection in another room can be caused by another person. Hence, room use duration is the length of time for which it is known with certainty that a room is being used and can therefore be shorter than the actual dwell time. In this study, room use duration for both 1- and 2-person homes was calculated using the same method (without looking at motion in other rooms in the homes), and results were therefore comparable between 1- and 2-person homes. If there was more than 1 room of the same type present in a home (eg, bedroom 1 and bedroom 2), only bedroom 1 (the main bedroom) was analyzed.
- TOOH: TOOH was calculated using the door contact sensor on egress doors and PIR motion sensors. It was defined as the time between 2 door status changes (open–close–open) when no motion was detected by the motion sensors during that time. For 2-person homes, this means that both residents had to leave the home before it was counted as TOOH.
- ILSA: ILSA was calculated using PIR motion sensors. It was defined as the time that 2 rooms in the home were used simultaneously, which means at least 2 people in the home using 2 different rooms. For 1-person homes, these people could be the participant and a visitor (overnight visitors were excluded as noted later) or a large pet.
- Percentage of rooms used: The percentage of rooms used was calculated using PIR motion sensors. It was defined as the number of rooms where motion was detected per day divided by the total number of rooms in the home. The total number of rooms in the home was defined as the total number of PIR motion sensors in the home based on the deployment protocol specifying that there was 1 PIR motion sensor installed in each room in the home.

### Data Processing

Days were excluded from analyses when the participant indicated on the weekly questionnaire that there were overnight visitors or the residents were away from home overnight and when 1 or more sensors were inactive (eg, due to a low battery). Furthermore, to avoid the influence of COVID-19–pandemic related restrictions, declared on March 20, 2020, in Illinois and on March 23, 2020, in Oregon, data collected after these dates for the respective cohorts were excluded. Moreover, if participants moved to a new home during the study or when they indicated that a person moved in or out of the home for longer than a month (eg, when relatives moved in or 1 of the partners passed away), data collected after the move or after the household resident change were excluded. To avoid the potential effect of participants behaving differently because they knew they were being monitored, the first 2 weeks’ data of each home were excluded from the analyses. Of the 232 homes included in CART [11], 209 (90.1%) had both complete clinical data and home sensor data. Of these 209 homes, 28 (13.4%) did not have sufficient data (>4 weeks of data) after excluding the days on which someone moved in or out of the home or the residents moved to a new home and after excluding the first 2 weeks of data.

We used 3 methods to compare 1-person homes with 2-person homes and MCI homes with no-MCI homes:

- Daily summaries: All outcome measures were calculated for each day per home and averaged over all the days afterward, leading to 1 outcome per outcome measure per home. Based on a trade-off between the number of homes with sufficient days of data collection and smaller variances (Figure S1 in Multimedia Appendix 1), we chose to take the mean of the first 4 weeks of eligible data from each home (after the exclusion of the first 2 weeks of data collection). Only weekdays were used for the analyses to avoid the effect of changing patterns during the weekend [16]. Data collection interruptions were ignored.
- Hour-to-hour summaries: All outcome measures were calculated for each hour for each day per home and averaged per hour across days afterward, leading to 24 outcomes for each outcome measure per home. Again, the first 4 weeks of eligible data and only weekdays were used for the analyses. This means that each hour for each participant was a representation of their average activity for that hour of the day over the 4-week study period.
- Daily change over time: All outcome measures were calculated for each day per home. The slope and variability were calculated for those daily measures. To capture meaningful changes, only homes that had a minimum measuring period of 6 months were included.

### Statistical Analysis

The 4 study groups (1-person MCI and no-MCI homes and 2-person MCI and no-MCI homes) were compared based on age, sex, and years of education using ANOVA, the Kruskal-Wallis test, or the chi-square test, as appropriate. Outliers deviating more than 5 SDs from the mean for each outcome variable were removed.

For daily summaries, linear models were used, with the daily summaries as the dependent variable and MCI status (MCI/no-MCI) and household-type (1-person/2-person) as independent variables, including the interaction effect between those variables, corrected for the mean age of the home, the number of females in the home, the number of males in the home, the number of White people in the home, the number of people of an ethnicity other than White in the home, the total number of rooms in the home, and the mean years of education of the home, according to the following model formula: PIR motion outcome ~ β_0_ + β_1_(2-person) + β_2_(MCI) + β_3_(2-person × MCI) + β_4_(age) + β_5_(females) + β_6_(males) + β_7_(White) + β_8_(other ethnicity) + β_9_(rooms) + β_10_(education).

Sex and ethnicity were treated as nominal variables, while age, rooms, and education were treated as continuous variables. The household type and MCI status were dummy variables (2-person vs 1-person and MCI vs no-MCI, respectively). To reduce the number of levels, only White and other ethnicities were used, as White was the most prevalent ethnicity in this sample. If the interaction effect of MCI status × household type was significant, analyses were stratified for household type, other the interaction effect was removed. For the percentage of rooms used, the covariate number of rooms was not included in the formula, as this was already included in the percentage of rooms used.

Hour-to-hour summaries were compared between groups using latent class trajectory analysis. The framework proposed by Lennon et al [17] was used to find the optimal model and the number of classes for each outcome measure separately using the *LCTMtools* and *lcmm* packages in R (R Core Team and the R Foundation for Statistical Computing). Models A (fixed effect: homoscedastic), B (fixed effect: heteroscedastic), C (random intercept), D (random slope), E (random quadratic: common variance structure across classes), F (random quadratic: proportionality constraint to allow variance structures to vary across classes), and G (random quadratic: unrestricted, class-specific variance structure) from Lennon et al [17] were tested, together with models H (model G but cubic instead of quadratic) and I (model G but quartic instead of quadratic). More detailed information about the models can be found in Table S1 from Lennon et al [17]. The chosen number of classes and model was based on the lowest Bayesian information criterion (BIC) among those models that converged. If there was a class with less than 2 homes (<1% according to Lennon et al [17]), a lower number of classes was chosen. Whether the household type and MCI status influenced in which class a home was classified was tested using multinomial logistic regression, again corrected for the mean age of the home, the number of females in the home, the number of males in the home, the number of White people in the home, the number of people of an ethnicity other than White in the home, the total number of rooms in the home, and the mean years of education of the home, with 1 model per outcome measure. The class with the largest group of homes assigned was chosen as the reference group: Class ~ β_0_ + β_1_(2-person) + β_2_(MCI) + β_3_(2-person × MCI) + β_4_(age) + β_5_(females) + β_6_(males) + β_7_(White) + β_8_(other ethnicity) + β_9_(rooms) + β_10_(education).

For the daily change over time, slopes and variances were compared using a linear model, with the slopes or variances as dependent variables and the MCI status and household type as independent variables and corrected for age. Only homes with more than 180 days (6 months) of data were included in the longitudinal analyses.

*P<*.05 was considered statistically significant. Statistical analyses were performed in R (V4.1.3).

### Ethical Considerations

All participants provided written informed consent before the start of the study. The study protocol was approved by the Oregon Health & Science Institutional Review Board (eIRB 17123), the Portland Veterans Affairs Institutional Review Board (IRB 4089), and the Rush University Institutional Review Board (16011407-IRB01).

## Results

### Participant Characteristics

We included 181 homes with more than 4 weeks of data after applying all the previously mentioned exclusion criteria, of which 129 (71.3%) were 1-person homes and 52 (28.7%) were 2-person homes (Table 1), leading to a total of 233 individual participants. The mean age and education were similar for all groups, but there were more females than males in 1-person no-MCI homes and more males than females in 1-person MCI homes. In 2-person homes, there was 1 home with a female+female couple, while all other homes included female+male couples. The mean age difference between 2 residents in 2-person homes was 2.9 (SD 5.7) years, with a maximum of 14.5 years. The majority of participants were White. The 2-person MCI homes had 2 residents, of which at least 1 resident had MCI, whereas in the 2-person no-MCI homes, neither resident had MCI. There were 5 (20.8%) 2-person MCI homes in which both residents were classified as having MCI, while the remaining 2-person MCI homes only had 1 resident classified as having MCI.

**Table 1:** Characteristics of the 4 study groups.

Characteristics			1-person homes				2-person homes		
			No MCI^a^ (n=102)		MCI (n=27)		No MCI (n=28)		MCI (n=24)
**Age (years), mean (SD)**			74 (6)		72 (6)		70 (4)		74 (7)
**Sex, n (%)**									
	Female	76 (74)		12 (44)		—^b^		—	
	Male	26 (26)		15 (56)		—		—	
	Female+male	—		—		27 (96)		24 (100)	
	Female+female	—		—		1 (4)		0 (0)	
**Education, mean (SD)**			15 (3)		15 (2)		15 (2)		14 (2)
**Race, n (%)**									
	White	59 (58)		22 (82)		—		—	
	Other	43 (42)		5 (18)		—		—	
	White+White	—		—		21 (75)		20 (84)	
	White+other	—		—		1 (4)		2 (8)	
	Other+other	—		—		6 (21)		2 (8)	
Number of rooms, mean (SD)			6 (3)		5 (3)		10 (3)		10 (3)
Follow-up (days), mean (SD)			640 (271)		631 (338)		898 (278)		943 (267)

^a^MCI: mild cognitive impairment.

^b^—: not applicable.

An overview and summary of all analyses results is presented in Table 2. Detailed results will be discussed in subsequent sections.

**Table 2:** Summary of all results.

Outcome measures and methods			2-person (vs 1-person homes)	MCI^a^ (vs no-MCI homes)		Was there a 2-person × MCI interaction effect?^b^
**TOOH^c^**						
	Daily summaries	Lower		—^d^	No	
	Hour-to-hour summaries	Lower all day		Lower at midday (in 2-person homes)	Yes	
	Daily change over time	—		—	No	
**ILSA^e^**						
	Daily summaries	Higher		Lower in 1-person homes, higher in 2-person homes	Yes	
	Hour-to-hour summaries	Higher all day		Lower in the afternoon (in 1-person homes), lower at night/in the morning (in 2-person homes)	Yes	
	Daily change over time	—		Higher change	No	
**Kitchen use**						
	Daily summaries	Higher		—	No	
	Hour-to-hour summaries	Higher at night, lower in the early morning		Higher at night (in 2-person homes)	Yes	
	Daily change over time	—		—	No	
**Bathroom use**						
	Daily summaries	—		—	Yes^f^	
	Hour-to-hour summaries	Lower in the evening/at night		Higher at night (in 2-person homes)	Yes	
	Daily change over time	—		Higher change (1-person only)	Yes	
**Living room use**						
	Daily summaries	—		—	No	
	Hour-to-hour summaries	Higher at night		—	No	
	Daily change over time	—		—	Yes^f^	
**Bedroom use**						
	Daily summaries	—		—	No	
	Hour-to-hour summaries	Higher at night		Lower value in the morning (in 2-person homes)	Yes	
	Daily change over time	—		Higher change	No	

^a^MCI: mild cognitive impairment.

^b^If an interaction effect was found, the analyses were stratified for household type.

^c^TOOH: time out of home.

^d^—: not applicable.

^e^ILSA: independent life space activity.

^f^Effects of the household type or MCI status disappeared after stratification for household type.

### Daily Summary Measures

**Figure 2:**
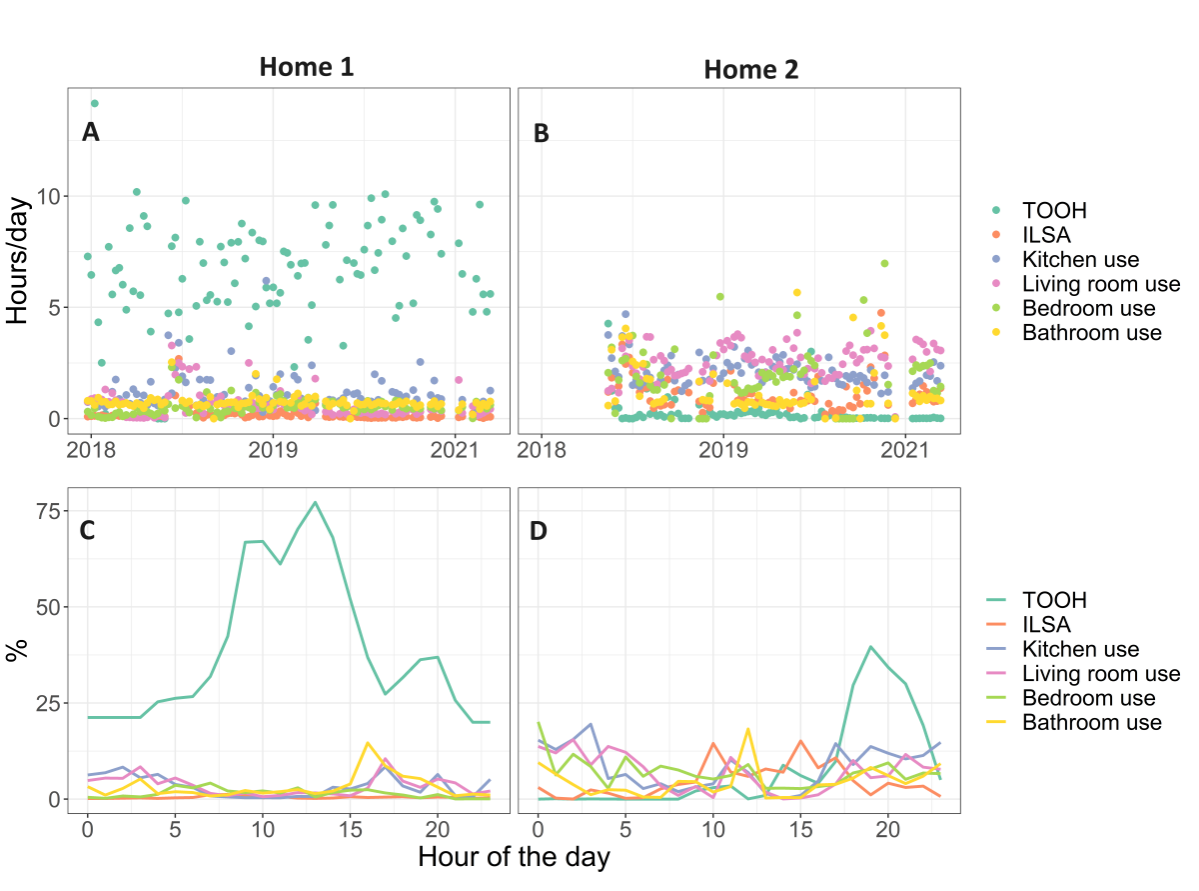
Data from 2 example homes. Home 1 (A and C) is a 1-person no-MCI home and home 2 (B and D) is a 2-person MCI home. (A and B) Change over time; each point represents 1 week. Home 2 was enrolled later in the study than home 1. Home 1 shows overall more TOOH, while home 2 shows more living room use. (C and D) Averaged hour-to-hour summaries, with variation over the day. The participant in home 1 leaves the house regularly during daylight hours, while the participants in home 2 leave the house usually during the evening. ILSA: independent life space activity; MCI: mild cognitive impairment; TOOH: time out of home.

**Figure 3:**
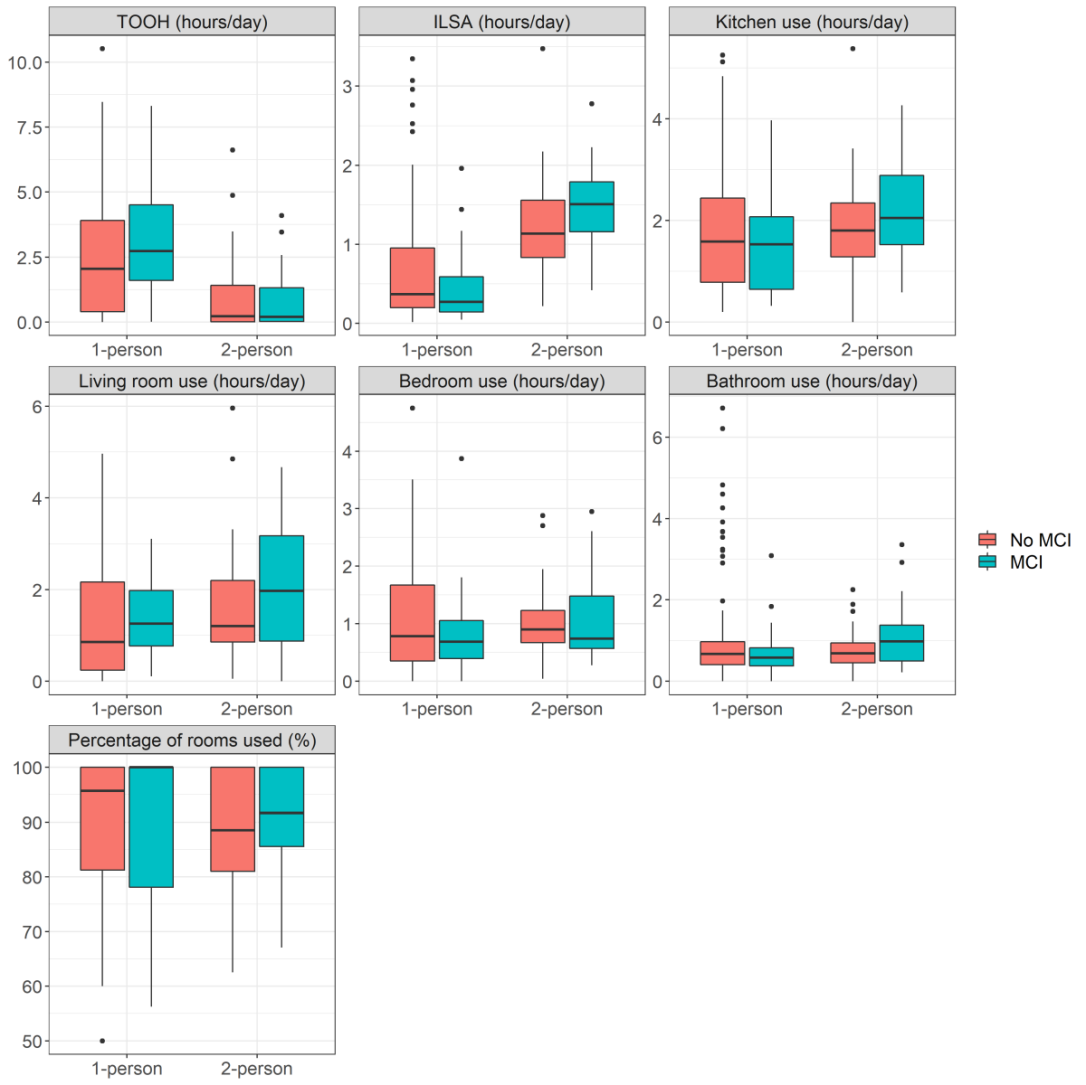
Boxplots from the life space activity metrics averaged over the first 4 weeks with data for 1- and 2-person homes and for MCI and no-MCI homes. ILSA: independent life space activity; MCI: mild cognitive impairment; TOOH: time out of home.

Data for 2 example homes are shown in Figures 2A and 2B (data for all homes can be found in Figure S2 in Multimedia Appendix 1). Overall, TOOH was shorter in 2-person homes than in 1-person homes (β=–2.8, SE 1.39, *P*=.047); see Figure 3. ILSA was longer in 2-person homes, with a significant interaction effect with MCI (β=0.59, SE 0.25, *P*=.02). After stratification for household type, in 1-person homes, MCI homes showed a shorter ILSA than no-MCI homes (β=–0.33, SE 0.16, *P*=.046) but a longer ILSA in 2-person homes (β=0.31, SE 0.18, *P*=.09). Highlighting that room use does not necessarily display the actual dwell time, because it only shows the duration for which someone is moving, excluding the time that someone is stationary in the room, kitchen use was longer in 2-person homes (β=1.60, SE 0.73, *P*=.03), independent of MCI status. A significant interaction effect for bathroom use was found (β=0.82, SE 0.40, *P*=.04), with a shorter time in the bathroom in 1-person MCI homes (β=–0.47, SE 0.28, *P*=.098) but a longer time in 2-person MCI homes (β=0.39, SE 0.22, *P*=.08), although the difference was not significant. No effects were found for bedroom use, living room use, and percentage of rooms used.

### Hour-to-Hour, Time-of-Day Summaries

**Figure 4:**
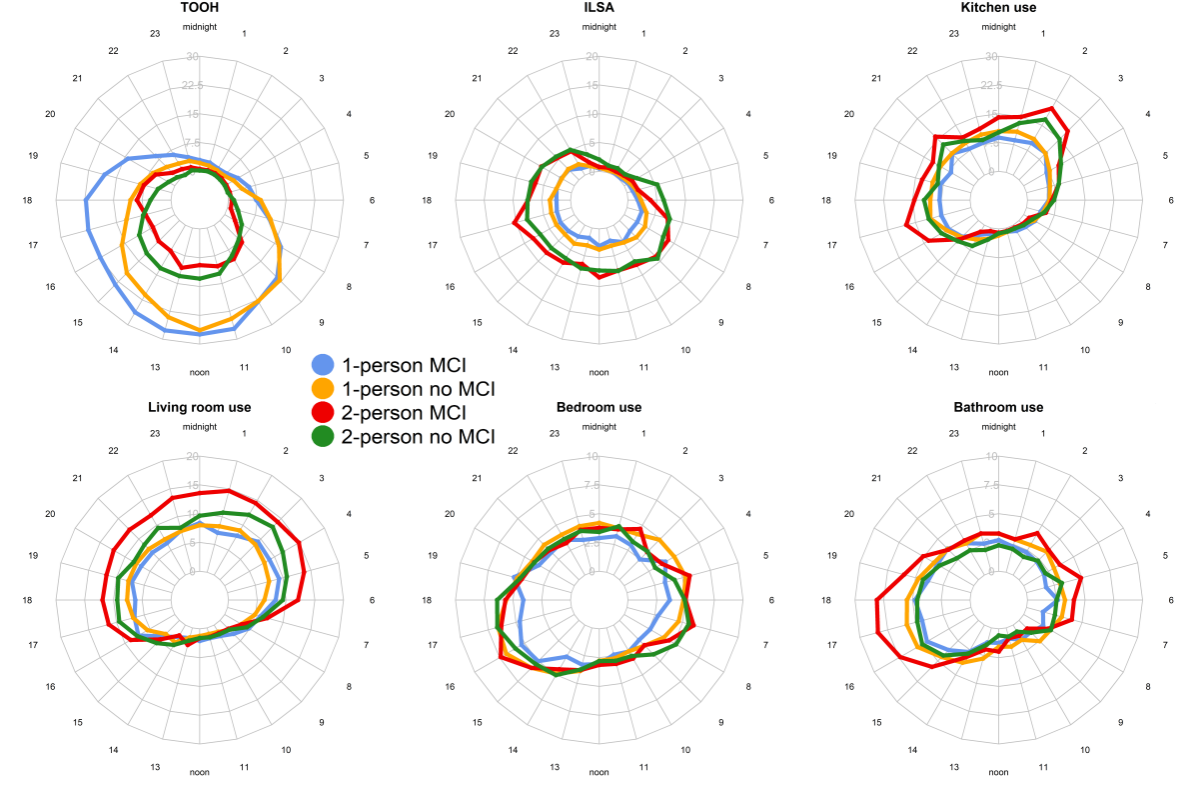
Spider plots with the group means for each study group for each life space activity metric. The hour-to-hour data were averaged over the first 4 weeks of data, including only weekdays. The axis represents percentage/hour for TOOH, ILSA, and room use in the kitchen, living room, bedroom, and bathroom. ILSA: independent life space activity; MCI: mild cognitive impairment; TOOH: time out of home.

Data for 2 example homes are shown in Figures 2C and 2D (data for all homes can be found in Figure S3 in Multimedia Appendix 1). Figure 4 shows the hour-to-hour group means. From this figure, it can be seen that TOOH was the highest in 1-person homes, with MCI homes showing the highest TOOH, even during the night. ILSA was higher in 2-person homes. Kitchen use was the highest in the early morning and late afternoon for 2-person homes, with the highest use in 2-person MCI homes. Living room use remained on the same level for the entire day for all homes. Bedroom use was the lowest for 1-person MCI homes for the entire day. Bedroom use was low at night because PIR motion sensors detect only motion and cannot detect stationary people (eg, when asleep). Bathroom use was similar for all groups, except for a large peak in the early morning and evening for 2-person MCI homes.

The chosen number of classes and best-fitted model per outcome measure are presented in Multimedia Appendix 2, together with the number of homes per class. The classes were determined for each outcome measure separately, meaning that the classes extracted for each outcome do not contain the same set of homes. Overall, 5 classes with model H (random cubic: unrestricted covariance structure) were found to be optimal, except for bathroom use, which identified 3 classes with model E (random quadratic: equal covariance structure). For TOOH and living room use, model I (random quartic: unrestricted covariance structure) showed the lowest BIC but did not converge, and therefore, model H with the second-lowest BIC was chosen. For all outcome measures, the class with the majority of homes was the “overall low” class, meaning that the majority of homes showed low values for each outcome measure during the entire day. Later, the results of the multinomial logistic regression models are discussed per outcome measure. A positive 2-person effect means that compared to 1-person homes, 2-person homes were more likely to follow the corresponding trajectory than the overall low trajectory. A positive MCI effect means that compared to no-MCI homes, MCI homes were more likely to follow the corresponding trajectory than the overall low trajectory. A positive interaction effect means that MCI homes were more likely to follow the corresponding trajectory in 2-person homes but less likely in 1-person homes. Classes with ≤5 homes assigned are not discussed to avoid potential accidental findings because of too few homes for informative analysis.

For TOOH, night high, midday high 1 and 2, and evening high trajectories were found, apart from the overall low trajectory. Overall, participants in 2-person homes were less likely to leave the home for the complete day (Table 3). Participants in 2-person MCI homes left the home less at midday, while participants in 1-person MCI homes left the home more at midday (interaction effect).

**Table 3:** Outcomes of the multinomial logistic regression models for TOOH^a^.

Class comparison	Trajectory	2-person homes		MCI^b^ homes			2-person × MCI interaction	
		OR^c^ (SE)	*P* value	OR (SE)	*P* value	OR (SE)		*P* value
1 vs 2 (n≤5)^d^	Night high	–19.62 (1.11)	<.001	–14.33 (1.11)	<.001	40.29 (1.11)		<.001
3 vs 2	Midday high 1	–15.40 (0.87)	<.001^e^	0.42 (0.64)	.51	–0.04 (1.10)		.97
4 vs 2	Evening high	–16.34 (0.93)	<.001^e^	–0.01 (0.63)	.98	–0.29 (1.47)		.84
5 vs 2	Midday high 2	–7.62 (0.00)	<.001^e^	0.47 (0.87)	.59	–3.70 (0.00)		<.001^e^

^a^TOOH: time out of home.

^b^MCI: mild cognitive impairment.

^c^OR: odds ratio.

^d^Classes with ≤5 people assigned are not discussed in the results.

^e^Significant results.

For ILSA, morning/night high and afternoon high 1, 2, and 3 trajectories were found, apart from the overall low trajectory. Participants living together were more likely to follow the afternoon high trajectory compared to participants living alone (Table 4). MCI homes were more likely to follow the afternoon high 3 trajectory in 2-person homes but less likely in 1-person homes. In addition, MCI homes were less likely to follow the morning/night high trajectory in 2-person homes but more likely in 1-person homes.

**Table 4:** Outcomes of the multinomial logistic regression models for ILSA^a^.

Class comparison	Trajectory	2-person homes		MCI^b^ homes			2-person × MCI interaction	
		OR^c^ (SE)	*P* value	OR (SE)	*P* value	OR (SE)		*P* value
2 vs 1	Afternoon high 1	–6.72 (0.83)	<.001^d^	–0.02 (0.58)	.97	–1.19 (1.62)		.46
3 vs 1	Morning/night high	0.92 (1.69)	.58	–0.89 (1.43)	.53	–23.80 (0.00)		<.001^d^
4 vs 1	Afternoon high 2	–5.47 (0.99)	<.001^d^	–0.50 (0.68)	.47	–0.17 (1.47)		.91
5 vs 1	Afternoon high 3	28.70 (0.64)	<.001^d^	–15.80 (0.72)	<.001^d^	14.61 (0.72)		<.001^d^

^a^ILSA: independent life space activity.

^b^MCI: mild cognitive impairment.

^c^OR: odds ratio.

^d^Significant results.

For kitchen use, night high 1 and 2, night/morning high, and evening high trajectories were found, apart from the overall low trajectory. Compared to 1-person homes, 2-person homes were less likely to follow the night high 1 trajectory and less likely to follow the night/morning high trajectory (Table 5). MCI homes were more likely to follow the night high 2 trajectory in 2-person homes but less likely in 1-person homes.

**Table 5:** Outcomes of the multinomial logistic regression models for kitchen use.

Class comparison	Trajectory	2-person homes		MCI^a^ homes			2-person × MCI interaction	
		OR^b^ (SE)	*P* value	OR (SE)	*P* value	OR (SE)		*P* value
1 vs 2	Night high 2	325.17 (1.61)	<.001^c^	–476.82 (0.76)	<.001^c^	478.95 (0.76)		<.001^c^
3 vs 2	Night high 1	–18.04 (0.73)	<.001^c^	0.03 (0.54)	.95	0.58 (0.94)		.54
4 vs 2	Night/morning high	–132.45 (0.99)	<.001^c^	0.56 (0.77)	.46	–0.39 (1.36)		.78
5 vs 2 (n≤5)^d^	Evening high	9.86 (0.00)	<.001	–116.94 (0.00)	<.001	–23.58 (0.00)		<.001

^a^MCI: mild cognitive impairment.

^b^OR: odds ratio.

^c^Significant results.

^d^Classes with ≤5 people assigned are not discussed in the results.

For bathroom use, night high and evening high trajectories were identified, apart from the overall low trajectory. MCI homes were more likely to follow the night high trajectory in 2-person homes but less likely in 1-person homes (Table 6). People living together were less likely to follow the evening high trajectory compared to people living alone.

**Table 6:** Outcomes of the multinomial logistic regression models for bathroom use.

Class comparison	Trajectory	2-person homes		MCI^a^ homes			2-person × MCI interaction	
		OR^b^ (SE)	*P* value	OR (SE)	*P* value	OR (SE)		*P* value
1 vs 2	Night high	–27.75 (0.65)	<.001^c^	–0.41 (0.72)	.56	16.42 (0.65)		<.001^c^
3 vs 2	Evening high	–3.99 (1.08)	<.001^c^	0.51 (0.97)	.60	0.62 (1.33)		.64

^a^MCI: mild cognitive impairment.

^b^OR: odds ratio.

^c^Significant results.

For living room use, afternoon/evening high and night high 1, 2, and 3 trajectories were found, apart from the overall low trajectory. The 2-person homes were more likely to follow the night high trajectory than the overall low trajectory compared to 1-person homes (Table 7).

**Table 7:** Outcomes of the multinomial logistic regression models for living room use.

Class comparison	Trajectory	2-person homes		MCI^a^ homes			2-person × MCI interaction	
		OR^b^ (SE)	*P* value	OR (SE)	*P* value	OR (SE)		*P* value
1 vs 4 (n≤5)^c^	Night high 3	197.04 (1.05)	<.001	–491.80 (0.90)	<.001	492.73 (0.90)		<.001
2 vs 4 (n≤5)^c^	Afternoon/evening high	112.95 (0.12)	<.001	–318.37 (0.12)	<.001	152.04 (0.12)		<.001
3 vs 4	Night high 1	89.21 (0.88)	<.001^d^	0.80 (0.61)	.19	–0.68 (1.05)		.52
5 vs 4	Night high 2	202.78 (0.45)	<.001^d^	–1.06 (1.25)	.40	1.52 (1.65)		.36

^a^MCI: mild cognitive impairment.

^b^OR: odds ratio.

^c^Classes with ≤5 people assigned are not discussed in the results.

^d^Significant results.

For bedroom use, the optimal number of classes was 5. However, when both 5 and 4 classes were chosen, this resulted in 1 class with n=1. Therefore, only 3 classes were chosen as the optimal number of classes. This resulted in evening high and night high trajectories, apart from the overall low trajectory. Only the night high trajectory had more than 5 homes and showed that 2-person homes were less likely to follow the night high trajectory compared to 1-person homes (Table 8).

**Table 8:** Outcomes of the multinomial logistic regression models for bedroom use.

Class comparison	Trajectory	2-person homes		MCI^a^ homes			2-person × MCI interaction	
		OR^b^ (SE)	*P* value	OR (SE)	*P* value	OR (SE)		*P* value
2 vs 1 (n≤5)^c^	Evening high	–11.51 (2.39)	<.001	–21.84 (0.00)	<.001	–0.63 (0.00)		<.001
3 vs 1	Night high	–4.65 (1.28)	<.001^d^	–0.76 (1.15)	.51	2.18 (1.79)		.22

^a^MCI: mild cognitive impairment.

^b^OR: odds ratio.

^c^Classes with ≤5 people assigned are not discussed in the results.

^d^Significant results.

### Daily Change Over Time

**Figure 5:**
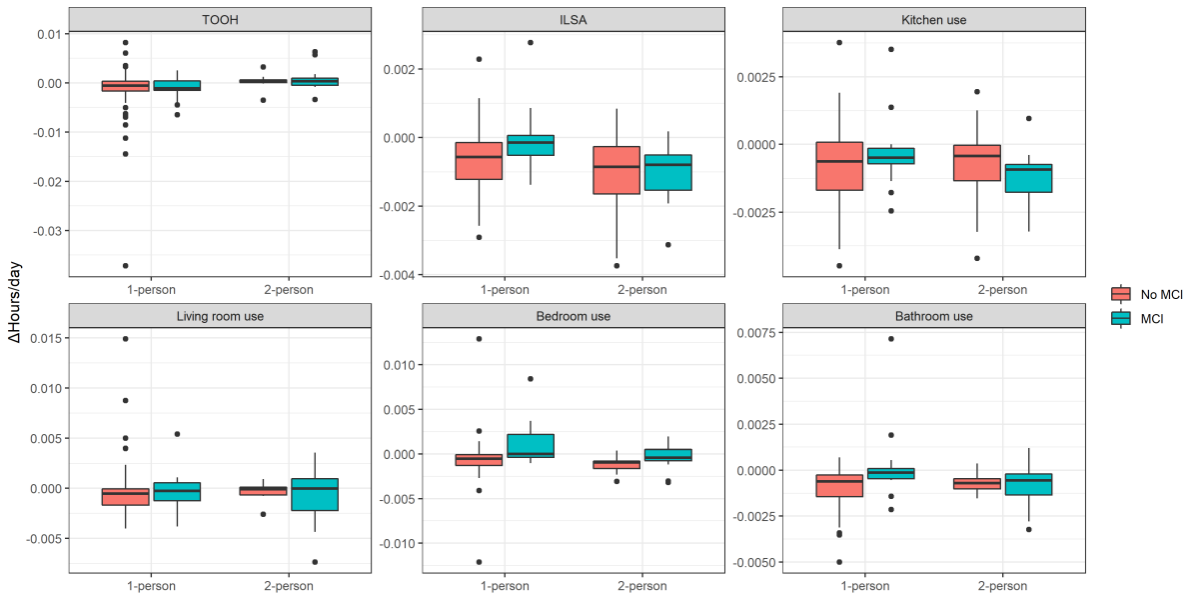
Boxplots of the slopes of the daily change over time for each outcome measure. ILSA: independent life space activity; MCI: mild cognitive impairment; TOOH: time out of home.

**Figure 6:**
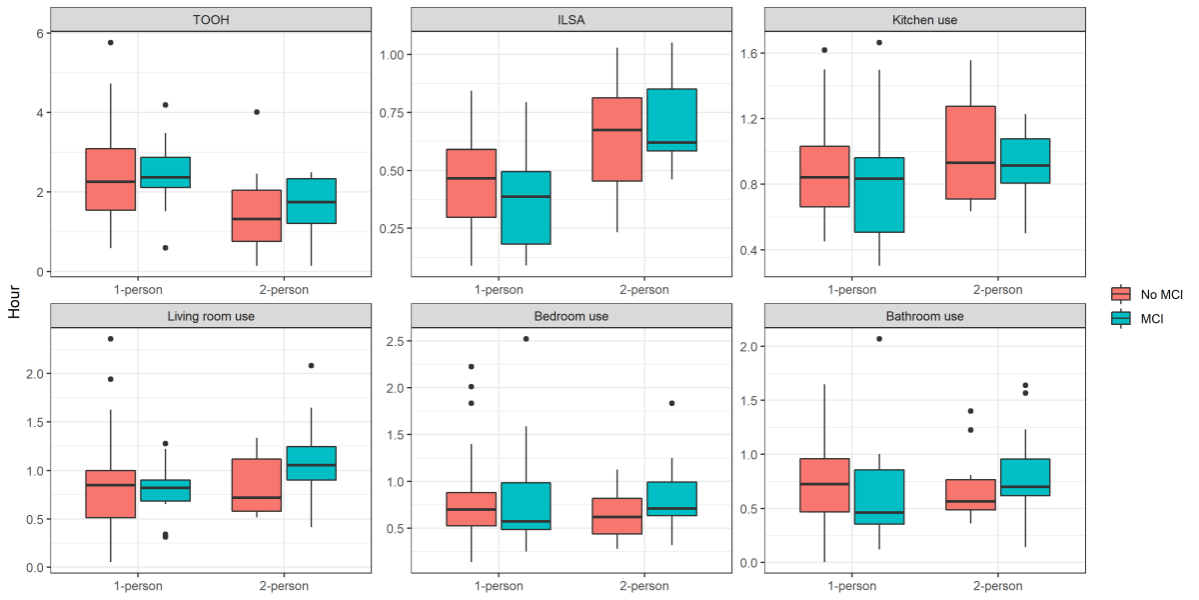
Boxplots of the variability (SD) of the daily change over time for each outcome measure. ILSA: independent life space activity; MCI: mild cognitive impairment; TOOH: time out of home.

After excluding homes with less than 180 days of data, we included 75 (n=16, 21.3%, MCI, n=59, 78.7%, no-MCI) 1-person homes and 28 (n=16, 57.1%, MCI, n=12, 42.9%, no-MCI) 2-person homes. The mean number of eligible days was 291 (SD 76) days. Differences in slopes were small (Figure 5): a greater change was found for MCI homes compared to no-MCI homes for ILSA (β=0.0006, SE 0.0002, *P*=.02) and bedroom use (β=0.002, SE 0.0006, *P*=.02), corresponding to 2.2 and 7.3 seconds per day and thus 13.4 and 44.4 minutes per year change, respectively. For bathroom use, an MCI status × household type interaction effect was found (β=0.0014, SE 0.0006, *P*=.03). After stratification for household type, only in the 1-person homes, MCI homes showed a greater change than no-MCI homes (β=0.002, SE 0.0005, *P*=.001). No other differences were found. For variability (Figure 6), an interaction effect was found for living room use (β=–0.45, SE 0.21, *P*=.04), but this effect disappeared when stratifying for household type. No other associations were found.

## Discussion

### Principal Findings

The aim of this study was to compare the everyday behavior patterns of people with MCI with those of cognitively normal participants using in-home passive sensors in both 1- and 2-person resident homes to determine whether there are differences in life activity patterns around the home that differ according to whether one lives alone and has MCI. TOOH, ILSA, bedroom use, bathroom use, living room use, kitchen use, and percentage of rooms used were analyzed in 3 ways: daily measures averaged over 4 weeks, hour-to-hour measures averaged over 4 weeks, and change over time. The most important outcome of this study is that the living situation of the participants was highly important when using objective measures, since we found that people living together have a shorter TOOH, a longer ILSA, and longer room use independent of analysis type. The effects of MCI status depended on whether someone was living alone or as a couple: In MCI homes, ILSA was, for example, lower in 1-person homes but higher in 2-person homes.

### Difference Between No-MCI and MCI Homes

When looking at daily measures, only ILSA was affected by MCI status: ILSA was higher in MCI homes compared to no-MCI homes in 2-person homes but lower in 1-person homes. This suggests that in 1-person homes, people without MCI have more visitors compared to people with MCI. This is according to expectations, as people with MCI tend to withdraw more from social activities [18]. In 2-person homes, a possible explanation for these findings is that a person without MCI living in a home with someone with MCI takes over more household duties, leading to more rooms being used at the same time. These results were confirmed by the hour-to-hour analyses.

To find the effects of MCI status on other outcome measures, hour-to-hour or longitudinal measures were needed: a greater change in ILSA, bedroom use, and living room use was seen in MCI homes. In the hour-to-hour analyses, the effects of MCI status were found on TOOH, ILSA, kitchen use, and bathroom use, but these effects depended on the household-type. These findings confirm what was already found by Wu et al [10,19]: hour-to-hour analyses and longitudinal changes over time need to be considered when one wants to find the effects of cognitive decline. A review by Yamasaki and Kumagai [20] of 10 studies that used in-home sensors to detect MCI also shows that especially day-to-day variability or a change in the time of day of activity patterns indicates cognitive decline. Our study adds to this evidence since this was also the case for 2-person homes, implying that the household type should be considered when analyzing activity data.

### Difference Between 1- and 2-Person Homes

Around 28% of the older population living in the United States in 2019 lived alone [21], meaning that 72% lived in a 2-or-more-person home. However, until now, almost all previous research using in-home unobtrusive objective measures has included people living alone [8-10], which is an important but different class within the general population. Our study showed that 1- and 2-person homes differ in terms of in-home everyday behavior patterns. All 3 analysis methods (daily summaries, hour-to-hour summaries, and change over time) showed effects of 1- and 2-person homes, which confirms that the household type is highly relevant when determining someone’s in-home behavior. To the best of our knowledge, this is the first study that shows the difference in life space activity patterns between people living alone and living as a couple.

The mean daily TOOH was higher in 1-person homes, which was expected, because in 2-person homes, 2 persons need to leave the home instead of 1 before it counts as TOOH. When looking at the hour-to-hour analyses, again, 1-person homes were more likely to follow trajectories with a high TOOH, especially at midday. Day-to-day variation was also higher in 1-person homes, meaning that TOOH varies more from day to day when someone is living alone instead of living with someone. ILSA was higher in 2-person homes, which was expected as well, since 1-person homes need a person to visit the home to have ILSA. This result was confirmed with the hour-to-hour analyses, where 2-person homes were also more likely to follow the high-ILSA trajectories independent of the time of day. We also found a greater change over time in 2-person homes compared to 1-person homes, but this change was small (13 minutes/year). The mean daily bedroom use did not differ between 1- and 2-person homes. The time-of-day analyses confirm the results of the daily measures as the majority of homes (88%) were in the class that followed the overall low trajectory. Bedroom use during the night was low, both in 1- and 2-person homes, which highlights the nature of PIR motion sensors: stationary people (eg, when sleeping) are not detected by the sensors. Bedroom use should therefore be interpreted as the time to prepare for bed or the day rather than the actual bedroom dwell time. The mean bathroom use did not differ between 1- and 2-person homes, which was unexpected, because it is unlikely that a person living in a 2-person home always uses the bathroom at the same time as their coresident. When looking at the hour-to-hour analyses, 1-person homes were more likely to use the bathroom at night compared to 2-person homes. The mean daily kitchen use was lower in 1-person homes than in 2-person homes, suggesting that people in 2-person homes spend more time preparing food.

Unexpected findings were that the kitchen was also used during the night by a large group (56% of the homes followed trajectories with kitchen use at night). These homes were more likely 2-person homes, suggesting that people in 2-person homes show more night-eating behavior. The kitchen is not always used only for eating purposes and can therefore also be used when someone is unable to sleep, implying that people in 2-person homes have more sleeping problems. These explanations are, however, speculations and should therefore be confirmed by follow-up research using various techniques, including selected direct visualization (eg, cameras), sleep sensors indicating poor sleep, and questionnaires (eg, the Ecological Momentary Assessment of sleep). Similar to kitchen activity, the mean living room use was lower in 1-person homes than in 2-person homes, and this was mainly seen in nighttime behavior: people in 2-person homes were more likely to use the living room around midnight, which again may suggest sleeping difficulties. A possible reason for this is that a person wakes up due to noises or movements from their partner. Further research with a bed mat, also incorporated in the CART data set [11], should confirm these findings.

### Strengths and Limitations

The strengths of this study are the passive, ecologically authentic data capture, a long follow-up time, a large sample size (>180 homes), and participants with different backgrounds and socioeconomic statuses. The fact that the 3 measuring methods converged in their results provides good evidence for the strength of the findings.

However, this study also has some limitations. First, for analysis of the daily change over time, we assumed that the change over time was linear, while change might be more complex, such as exponential or first marked by a slow change and then, after a change point, by a more precipitous increase or decrease. Second, the decision to omit the first 2 weeks of data to avoid the potential effect of being self-conscious of the activity about the home, which is being “observed” by the sensors, was made arbitrarily: further research should confirm that the potential observation effect receded after 2 weeks. Third, the effect of pets was not considered. When pets are large (eg, a large dog), they could activate the PIR motion sensors when no one is using the room or is even out of the house. However, the expected findings that TOOH was lower and ILSA was higher in 2-person homes suggest that this is not a major issue in these data. Fourth, the designation of MCI or dementia is a static state label given until a subsequent clinical assessment is performed that suggests a new diagnostic milestone has been reached. However, these designations may be unstable during 2-3 years of follow-up, with 4%-55% people reverting from MCI to normal cognition [22]. In this context, we note that there were 19 homes where a resident transitioned from normal cognition to MCI or from MCI to dementia during the study. We did not consider these changes, as there were too few cases in the different groups to be included in the analysis. It will be interesting to investigate in future research the change in life space activity patterns in different resident settings where a person transitions to MCI and dementia. Lastly, the measures we chose for this study are just illustrations to show that the household type can have an effect and are not meant to be an exhaustive list of examples.

### Future Research and Clinical Applicability

Future research should combine the outcome measures into 1 composite score to quantify overall in-home behavior. Adding continuous information, such as individual mobility (eg, steps on an actigraph) or sleeping information (eg, time in bed or out of bed from a bed mat), could improve the results. Furthermore, a longer follow-up duration would help model which behavior could possibly predict a conversion to dementia. Finally, examining specific activities more closely would enlarge our knowledge of which particular in-home activities are performed at a particular time, for example, if the kitchen used for cooking, the bedroom for sleeping, and the bathroom for showering. After these clarifications, the results of this study can be used to monitor people more sensitively, continuously, at home, and without the need for active interaction from the participants. This can, for example, be used in a clinical trial or as a screener for needed assistance.

### Conclusion

This study demonstrated that life space activity patterns, as measured with passive in-home sensors, are influenced by both the household type and the MCI status. This confirms that changed in-home behavior can be seen if 1 person in the home has MCI, even if the sensors cannot distinguish between residents. To show the influence of MCI status, data should be analyzed as time-of-day changes or longitudinal changes. Future research needs to consider the household type, as 2-person homes show different behavior than 1-person homes and this can affect the clinical assessment of functional activity patterns unique to those experiencing cognitive decline over time.

## Appendix

Multimedia Appendix 1 Supplementary figures.

Multimedia Appendix 2 Chosen number of classes and best-fitted model per outcome measure.

## Abbreviations

BIC: Bayesian information criterion
CART: Collaborative Aging Research Using Technology
CDR: Clinical Dementia Rating
ILSA: independent life space activity
MARS: Minority Aging Research Study
MCI: mild cognitive impairment
OHSU: Oregon Health & Science University
PIR: passive infrared
RUSH: Rush University Medical Center
TOOH: time out of home
