# Supplementary material for: Differences in Life Space Activity Patterns Between Older Adults With Mild Cognitive Impairment Living Alone or as a Couple: Cohort Study Using Passive Activity Sensing
Source: JMIR Aging. 2023 Oct 11;6:e45876. doi: 10.2196/45876 (PMC10600648; doi:10.2196/45876)
Supplement: Multimedia Appendix 1 [file aging_v6i1e45876_app1.docx]

# Multimedia Appendix 1


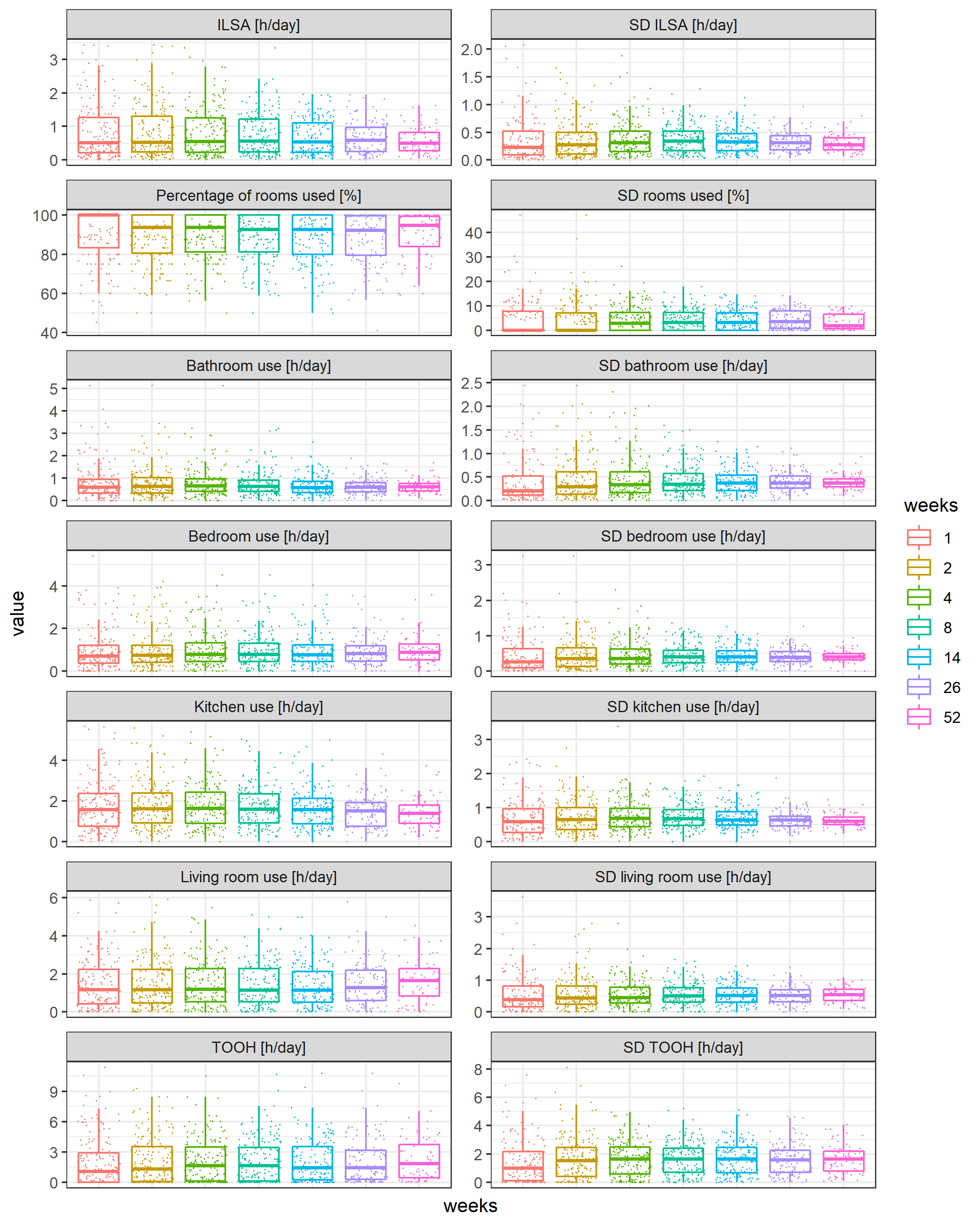


**Supplementary Figure 1** – means of median and standard deviation of independent life space activities (ILSA), percentage rooms used, room use of bathroom, bedroom, kitchen, and living room in hours, and time out of home (TOOH) averaged over 1, 2, 4, 8, 14, 26 and 52 weeks, for weekdays only. Each point represents 1 home. Only homes that have data for the x number of weeks are included, resulting in n=181 for 1 week, n=181 for 2 weeks, n=178 for 4 weeks, n=175 for 8 weeks, n=162 for 14 weeks, n=128 for 26 weeks and n=72 for 52 weeks.


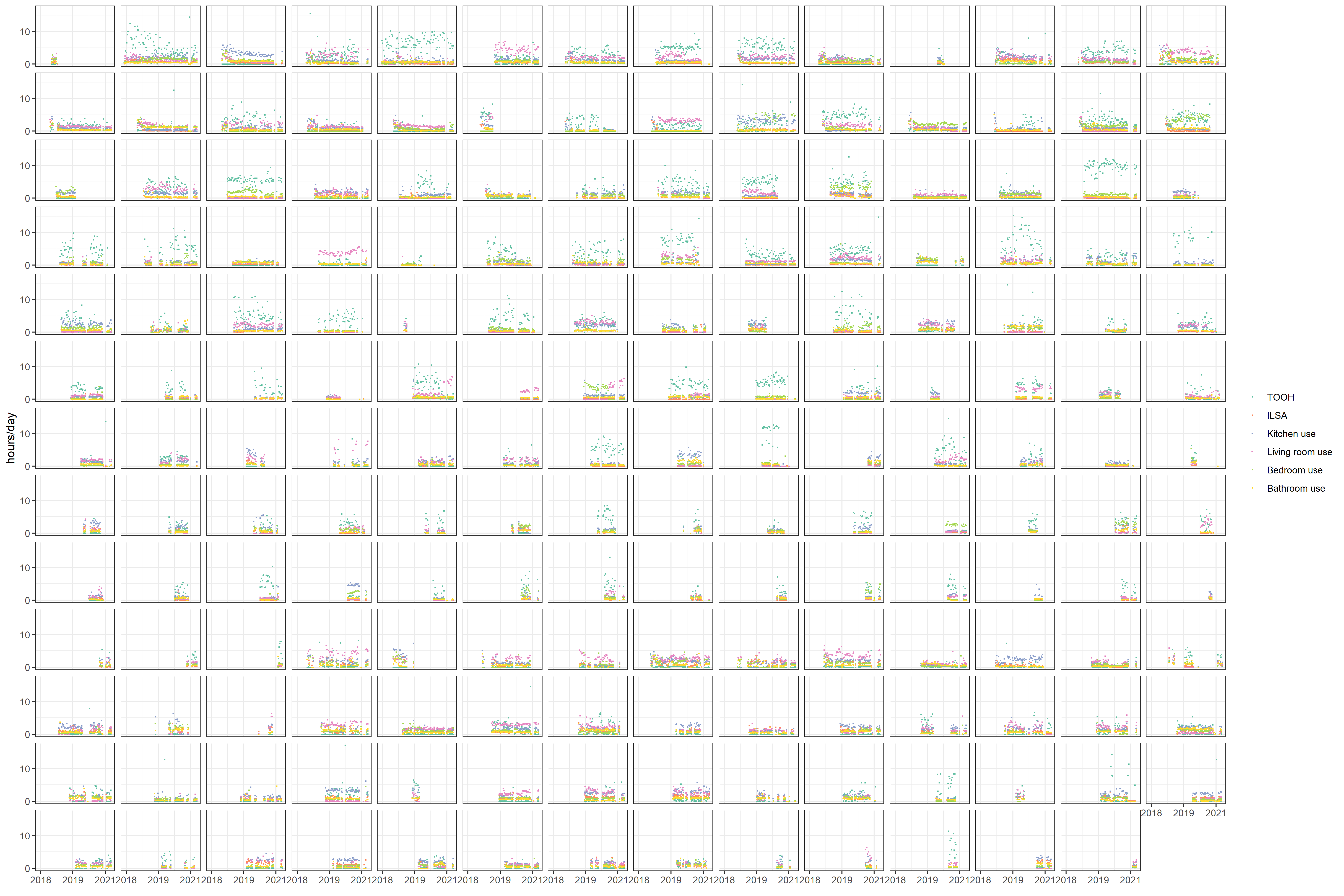


**Supplementary Figure 2** – Weekly values for the PIR motion outcome measures per home. Every point represents one week. Abbreviations: TOOH = Time Out of Home, ILSA = Independent Life Space Activities.


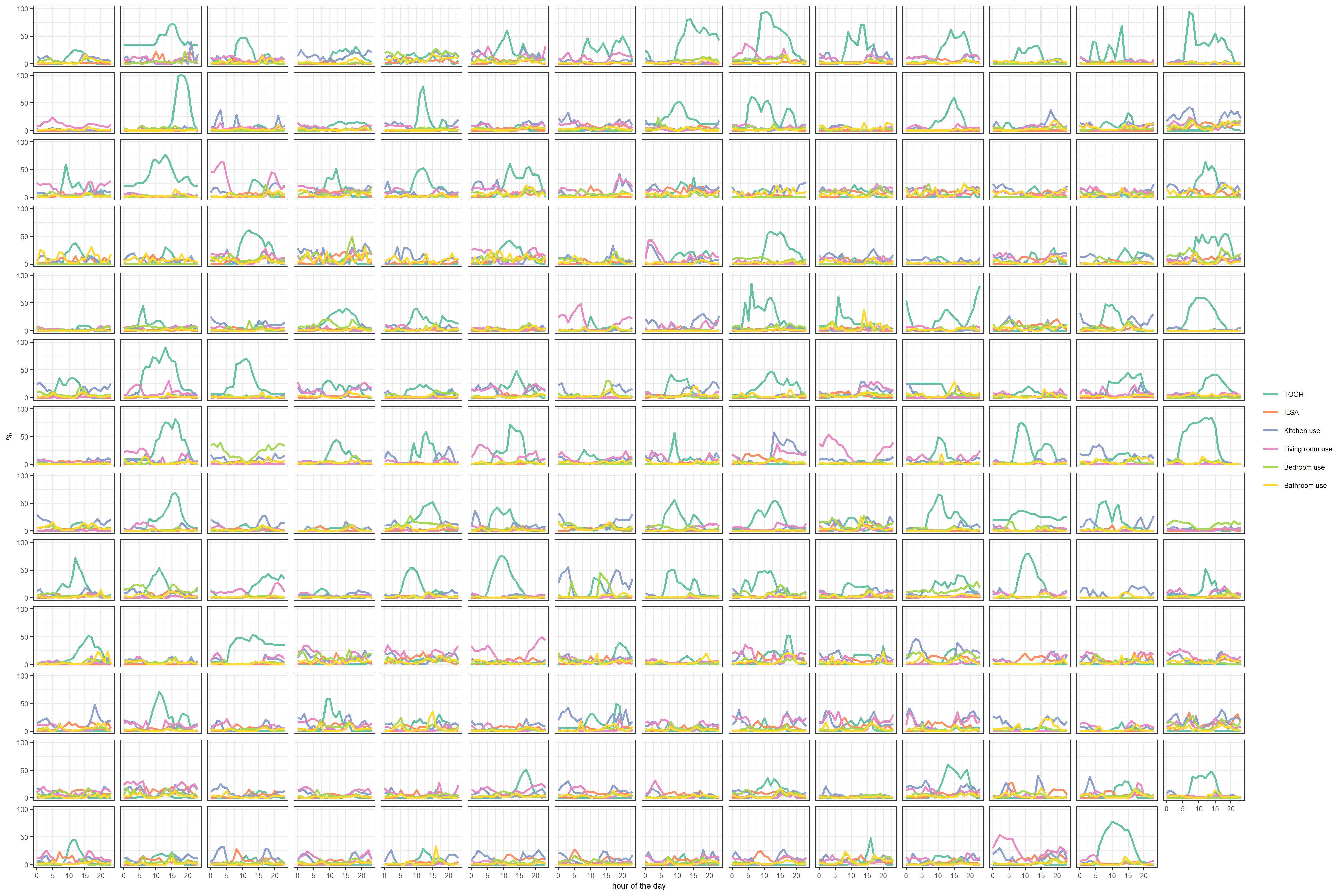


***Supplementary Figure 3*** *– Hour-to-hour data was averaged over the first 4 weeks of data with weekdays only. The y-axis displays %, with 100 meaning the complete hour spent in the kitchen, living room, bedroom, bathroom, out of the home (TOOH) or during independent life space activities (ILSA).*
