# Supplementary material for: Differences in Life Space Activity Patterns Between Older Adults With Mild Cognitive Impairment Living Alone or as a Couple: Cohort Study Using Passive Activity Sensing
Source: JMIR Aging. 2023 Oct 11;6:e45876. doi: 10.2196/45876 (PMC10600648; doi:10.2196/45876)
Supplement: Multimedia Appendix 2 [file aging_v6i1e45876_app2.docx]

**Table S1.** Hour-to-hour trajectory classes of time-of-day-based activity and the number of homes per class. Green shading indicates the dominant trajectory pattern for each activity type. Model H (random cubic: unrestricted covariance structure) and model E (random quadratic: equal covariance structure).

|  |  | 1-person homes | | 2-person homes | |  |  |
| --- | --- | --- | --- | --- | --- | --- | --- |
| Class | **Class name** | **MCI^a^ (N)** | **No-MCI (N)** | **MCI (N)** | **No-MCI (N)** |  |  |
| TOOH^b^ (model H) | | | | | | | |
| 1 | Night high | 0 | 3 | 1 | 0 | 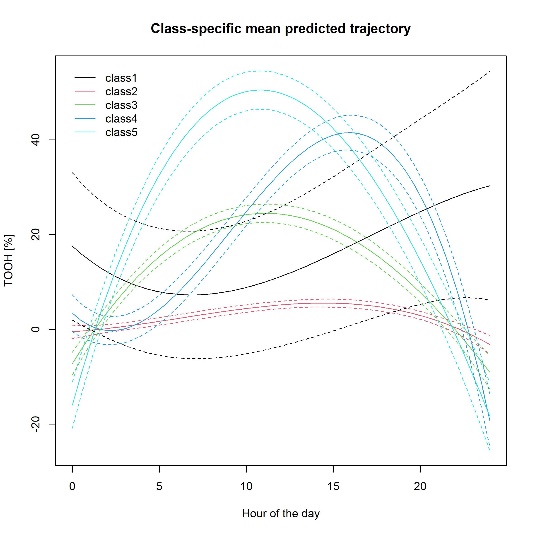 |  |
| 2 | Overall low | 10 | 45 | 19 | 22 |  |  |
| 3 | Midday high 1 | 6 | 27 | 3 | 3 |  |  |
| 4 | Evening high | 8 | 17 | 1 | 2 |  |  |
| 5 | Midday high 2 | 3 | 10 | 0 | 1 |  |  |
| ILSA^c^ (model H) | | | | | | | |
| 1 | Overall low | 15 | 50 | 2 | 2 | 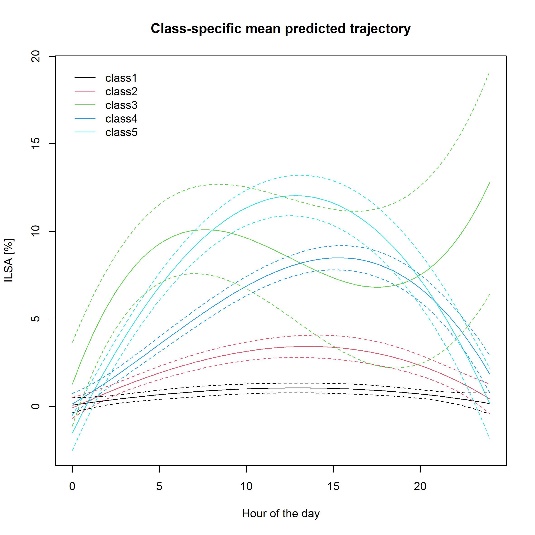 |  |
| 2 | Afternoon high 1 | 7 | 26 | 2 | 4 |  |  |
| 3 | Morning/ night high | 1 | 3 | 0 | 3 |  |  |
| 4 | Afternoon high 2 | 4 | 19 | 12 | 12 |  |  |
| 5 | Afternoon high 3 | 0 | 4 | 8 | 7 |  |  |

| Kitchen use (model H) | | | | | | | |
| --- | --- | --- | --- | --- | --- | --- | --- |
| 1 | Night high 2 | 0 | 6 | 5 | 1 | 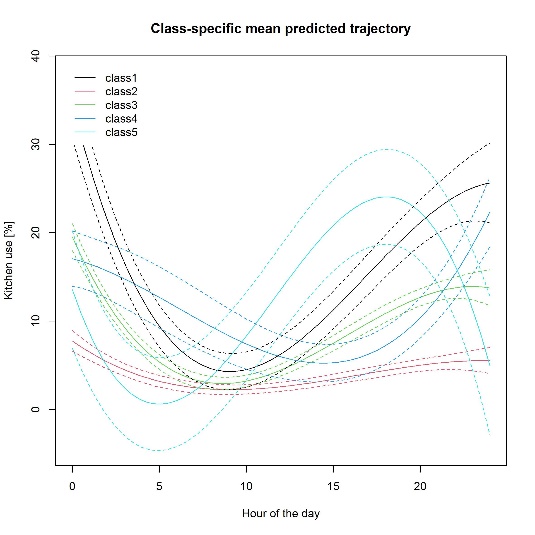 |  |
| 2 | Overall low | 13 | 49 | 4 | 9 |  |  |
| 3 | Night high 1 | 9 | 33 | 13 | 15 |  |  |
| 4 | Night / morning high | 4 | 9 | 2 | 3 |  |  |
| 5 | Evening high | 1 | 3 | 0 | 0 |  |  |
| Bathroom use (model H) | | | | | | | |
| 1 | Night high | 3 | 17 | 5 | 0 | 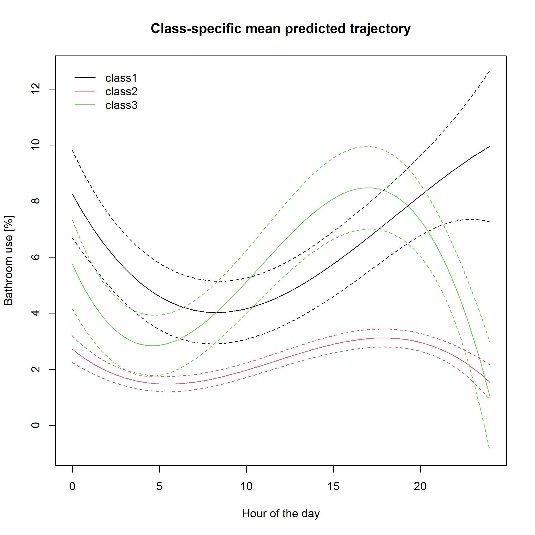 |  |
| 2 | Overall low | 21 | 74 | 15 | 25 |  |  |
| 3 | Evening high | 2 | 10 | 4 | 3 |  |  |

| Living room use (model H) | | | | | | | |
| --- | --- | --- | --- | --- | --- | --- | --- |
| 1 | Night high 3 | 0 | 3 | 1 | 1 | 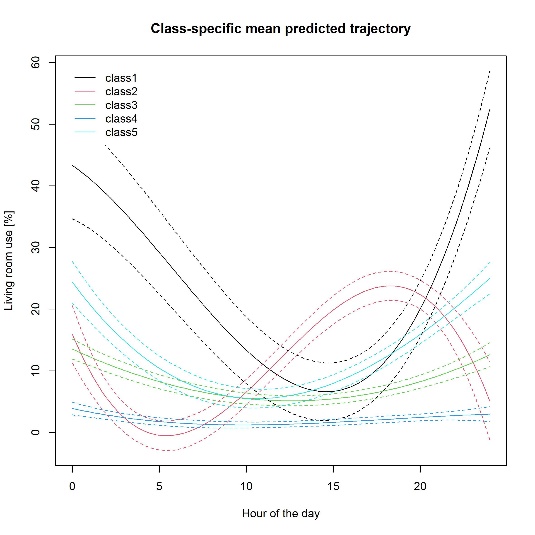 |  |
| 2 | Afternoon / evening high | 0 | 2 | 0 | 0 |  |  |
| 3 | Night high 1 | 14 | 30 | 10 | 12 |  |  |
| 4 | Overall low | 9 | 50 | 5 | 8 |  |  |
| 5 | Night high 2 | 1 | 9 | 8 | 5 |  |  |
| Bedroom use (model E) | | | | | | | |
| 1 | Overall low | 23 | 84 | 22 | 25 | 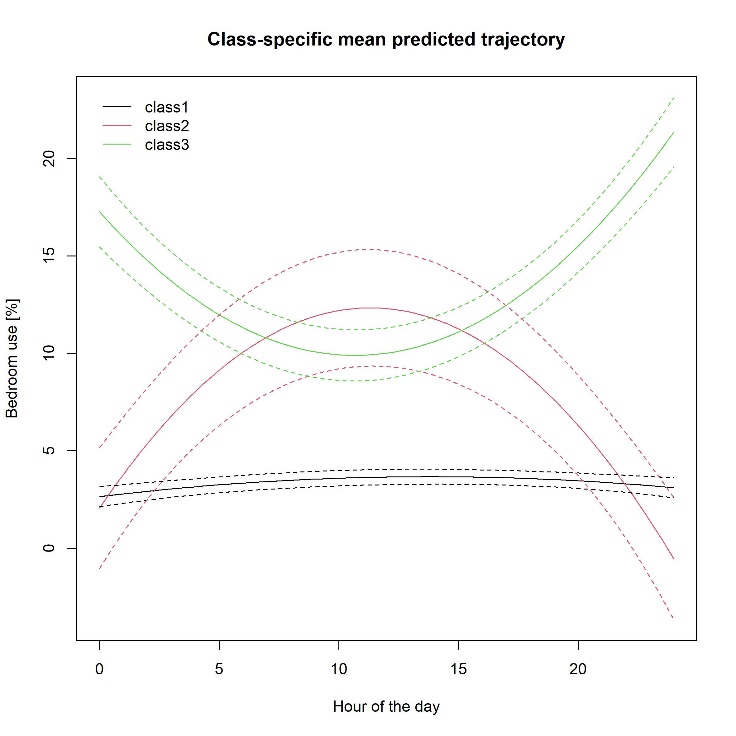 |  |
| 2 | Midday high | 0 | 3 | 0 | 1 |  |  |
| 3 | Night high | 1 | 10 | 2 | 1 |  |  |

^a^MCI: mild cognitive impairment.

^b^TOOH: time out of home.

^c^ILSA: independent life space activity.
